# Supplementary figures and images for: Intoxication of antibiotic persisters by host RNS inactivates their efflux machinery during infection
Source: PLoS Pathog. 2024 Feb 29;20(2):e1012033. doi: 10.1371/journal.ppat.1012033 (PMC10903880; doi:10.1371/journal.ppat.1012033)

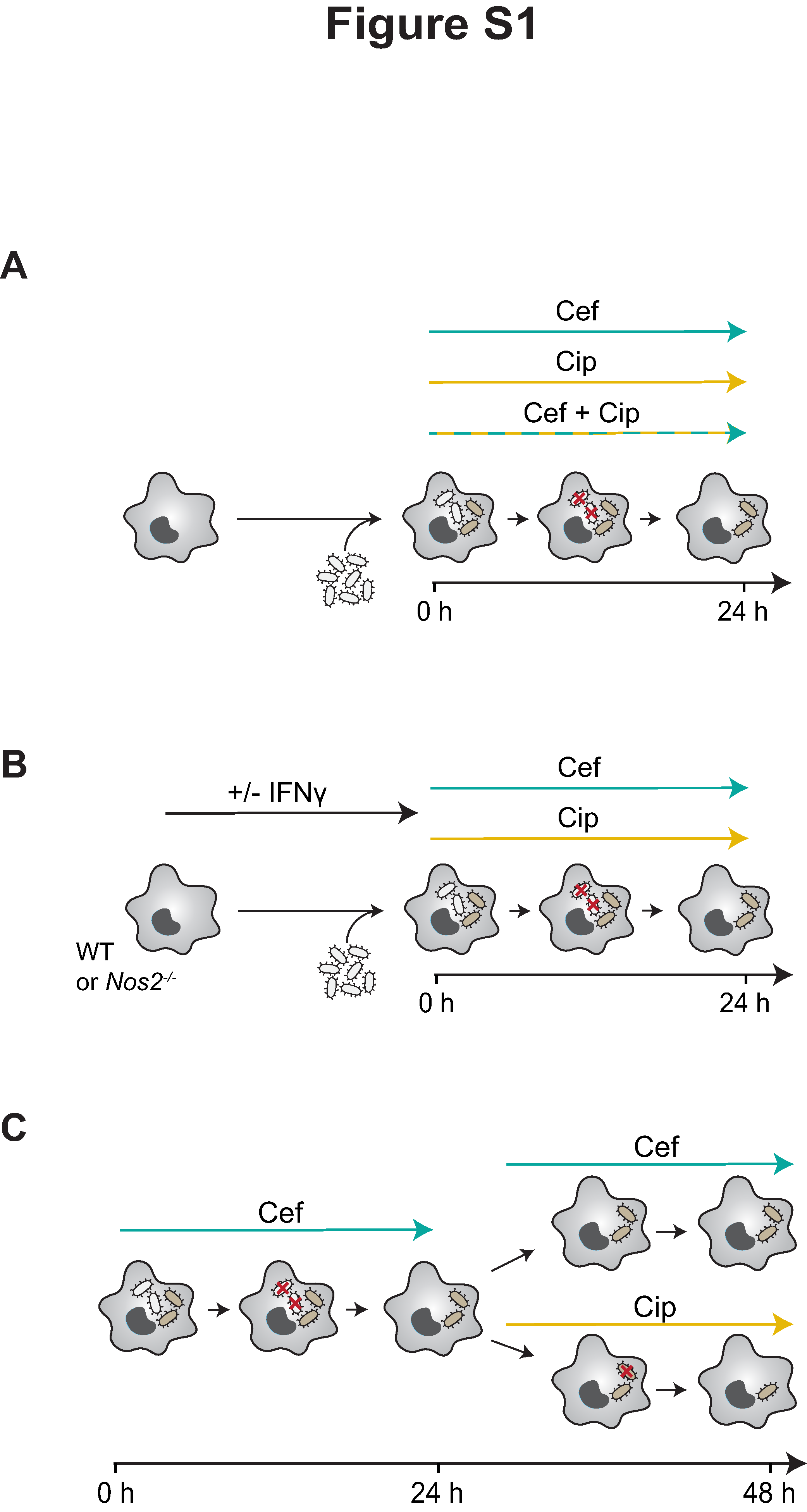

Supplement: S1 Fig — (A) Bone marrow-derived macrophages from WT mice were infected with WT Salmonella. Then, infected Mφ were treated with cefotaxime, ciprofloxacin or both for 24 h. Finally, persisters (in brown) were extracted and plated on LB agar plate for counting. (B) Bone marrow-derived macrophages from WT or Nos2-/- mice were cultivated in the absence or in the presence of IFN-γ and infected with WT Salmonella. Then, infected Mφ were treated with cefotaxime or ciprofloxacin for 24 h. Finally, persisters (in brown) were extracted and plated on LB agar plate for counting. (C) Infected bone marrow-derived macrophages from WT mice were treated for 24 h with cefotaxime to select persisters. Then, infected macrophages were treated with cefotaxime or ciprofloxacin for 24 additional hours. Finally, persisters (in brown) were extracted and plated on LB agar plate for counting. (TIF) [file ppat.1012033.s001.tif]

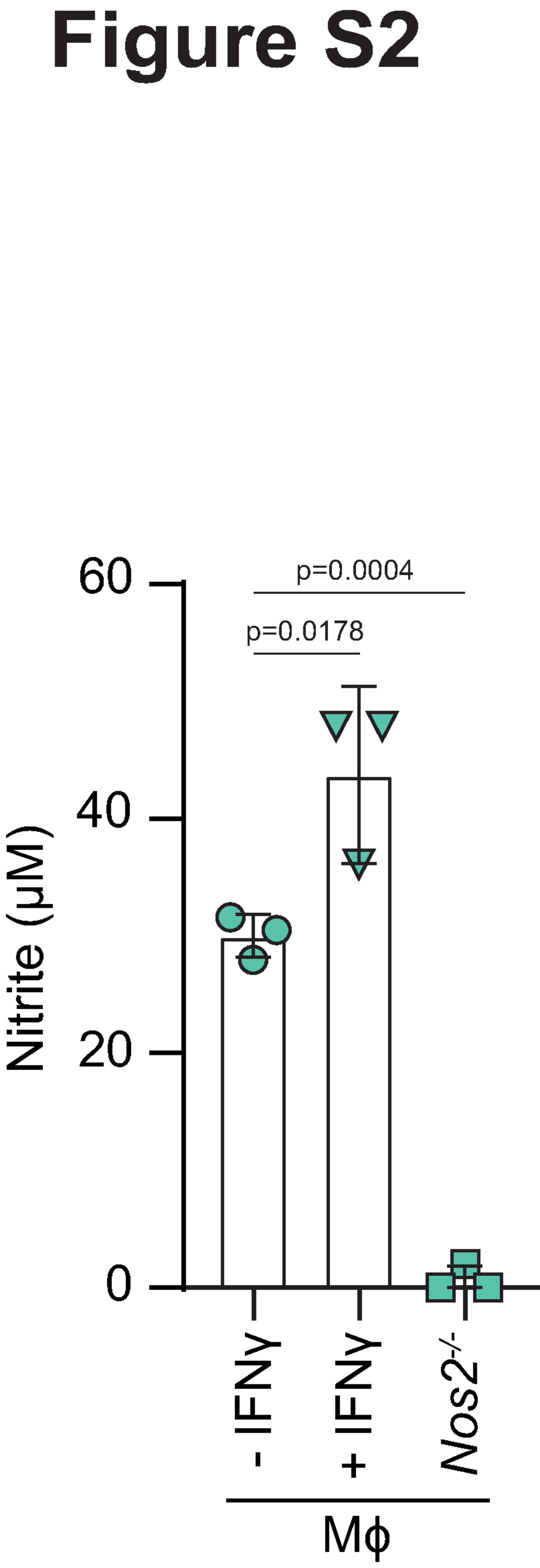

Supplement: S2 Fig — Quantification of nitric oxide production by macrophages was achieved by quantifying its stable byproduct nitrite in the infection medium. Nitrite concentration in the infection medium of unstimulated (circle) or IFN-γ-stimulated (triangle) WT and Nos2-/- (square) Mφ infected for 24 h with WT Salmonella and treated with cefotaxime. p values are indicated (ANOVA with Dunnett’s correction for multiple testing against the—IFN- γ condition); error bars depict means and standard deviation (SD); n = 3. (TIF) [file ppat.1012033.s002.tif]

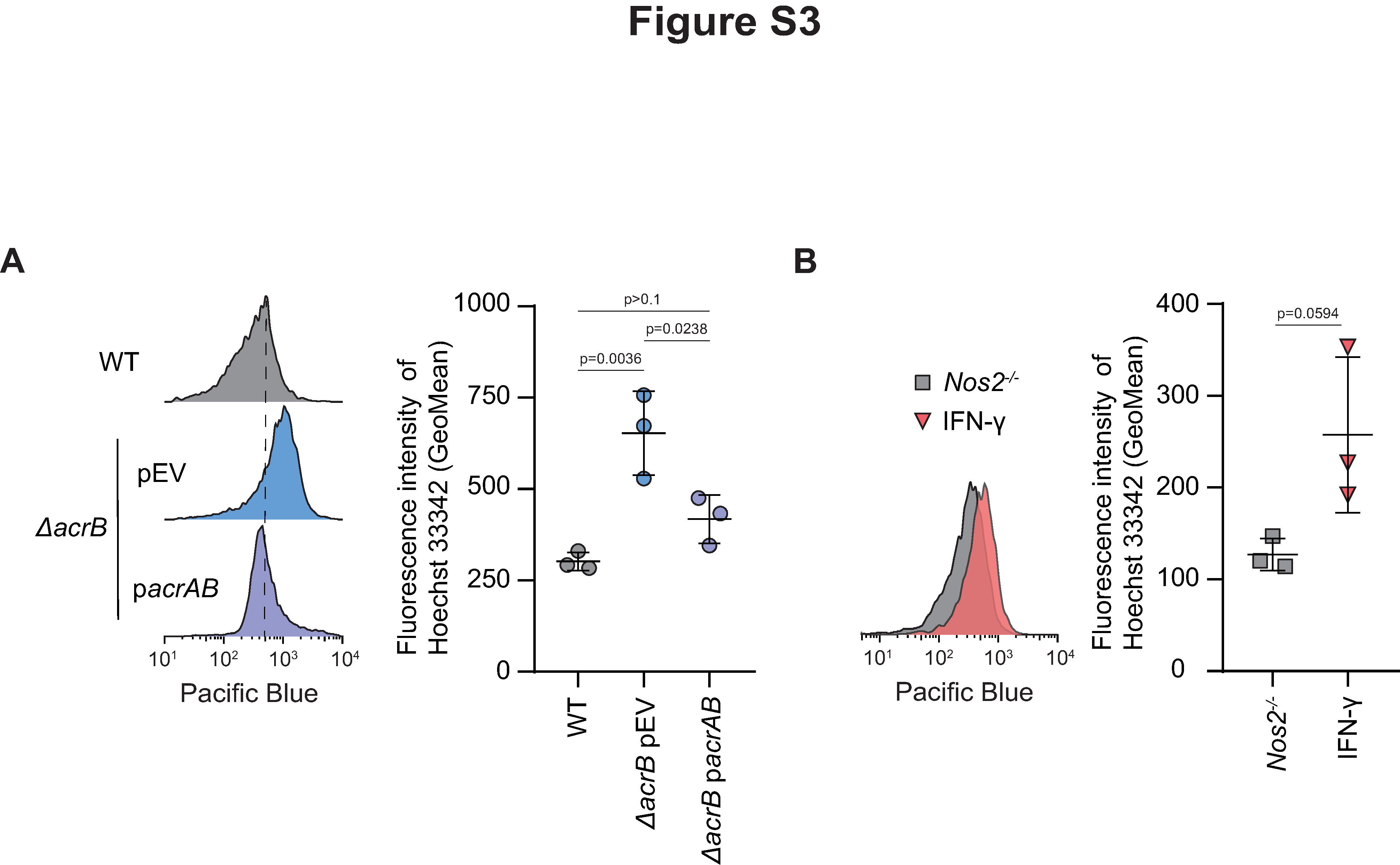

Supplement: S3 Fig — (A) Representative FACS plots and quantification of the efflux activity of WT or ΔacrB complemented with an empty vector (pEV) or acrAB (pacrAB) treated with 2 μM H33342 dye. p values are indicated (ANOVA with Tukey’s correction for multiple comparisons); error bars depict means and standard deviation (SD); n = 3. Experimental conditions are the same as in Fig 3D. (B) Representative FACS plots and quantification of the efflux activity of WT Salmonella in Nos2-/- (gray) or IFN-γ-stimulated WT (red) Mφ. p value is indicated (unpaired t test); error bars depict means and standard deviation (SD). Experimental conditions are the same as in Fig 3F. (TIF) [file ppat.1012033.s003.tif]
