# Supplementary material for: Intoxication of antibiotic persisters by host RNS inactivates their efflux machinery during infection
Source: PLoS Pathog. 2024 Feb 29;20(2):e1012033. doi: 10.1371/journal.ppat.1012033 (PMC10903880; doi:10.1371/journal.ppat.1012033)
Supplement: Attachment — Submitted filename: Ronneau et al review.docx [file ppat.1012033.s007.docx]

The manuscript by Ronneau S. *et al.* explores the mechanism underlying a recent observation, that reactive nitrogen species (RNS) sensitizes *Salmonella* to fluoroquinolones. They show RNS targeting of the TCA cycle results in reduction of proton-motive force, preventing ciprofloxacin efflux from the bacterial cell. While ciprofloxacin accumulation was not measured directly, the authors utilized multiple approaches to detect efflux activity and correlate the reduction in efflux activity with ciprofloxacin sensitivity. They also utilized a complemented strain to show altered sensitivity is due to efflux. They incorporated clinical isolates into experiments as well, to show the relevance of these pathways for clinical strains.

The findings here provide an important advance in our understanding of antibiotic tolerance, and combating antibiotic tolerance cells, and the authors systemically explore several different aspects of their model through each Figure. The experiments are well-designed, and strongly support the authors conclusions. I have one suggestion for an additional experiment below with the *acrAB* complemented strain that I think could strengthen their model further, however the authors have shown the more critical complementation of the survival defect with this strain. This manuscript is extremely well-written, and I only have minor comments below:

Specific comments:

- Since the authors have generated a complemented strain (∆*acrB* + p*acrAB*, in Fig. 3G), it would also be important to show this strain no longer has increased accumulation of H33342. It was great to see the authors utilize this strain to complement the survival phenotype, but I think also showing the impact on H33342 accumulation should be included.
- Fig. 2E: The GFP intensity quantification is described as ‘translational activity’, but really would be a combination of transcription, translation, fluorescent protein folding, and fluorophore maturation. I think a term like ‘Induction capacity’ or ‘Induction ability’ might more accurately reflect GFP intensity here.
- Fig. 4: The model described here was cited briefly once in the text, and could have been discussed in more detail. Some discussion is already present, and filling in additional ‘Fig. 4’ references in the text would reinforce this by drawing the reader to the Figure. The authors should also consider adding a couple of additional sentences to reinforce some of the ideas in Fig. 4B, particularly the heterogeneity of efflux expression that is depicted here.
- Lines 219-220, Fig. 3: The concentrations of H33342 listed in the Results text (0, 0.2, 1, 2) don’t match the concentrations shown in Fig. 3F (0, 0.2, 0.5, 1). H33342 is written as ‘H33345’ in Fig. 3D and 3F.
- Minor: line 262, missing ‘damage’ after DNA?
- Minor: typo line 173, ‘onlybe’
