# Supplementary material for: Intoxication of antibiotic persisters by host RNS inactivates their efflux machinery during infection
Source: PLoS Pathog. 2024 Feb 29;20(2):e1012033. doi: 10.1371/journal.ppat.1012033 (PMC10903880; doi:10.1371/journal.ppat.1012033)
Supplement: Attachment — Submitted filename: Ronneau Efflux revisions Response to reviewers.docx [file ppat.1012033.s008.docx]

**PPATHOGENS-D-23-01758 - [EMID:38f0e7e59950073d]**

**Intoxication of Antibiotic Persisters by Host RNS Inactivates their Efflux Machinery During Infection**

Séverin Ronneau, Charlotte Michaux, Rachel T. Giorgio and Sophie Helaine

**Reviewer #1**

In this impressive and detailed study, the authors set out to investigate a puzzling observation – release of reactive nitrogen species (RNS) in macrophages increases production of persisters tolerant of b-lactams in intracellular Salmonella, but these persisters are vulnerable to a fluoroquinolone. RNS inhibit respiration, which causes partial dormancy, inhibits growth, and explain tolerance of b-lactams. The authors now show that the same decrease in energy, specifically pmf, diminishes the activity of the AcrAB/TolC MDR pump, which increases the level of ciprofloxacin in the cell, “intoxicating” persisters. Specific comments follow.

We thank the reviewer for their very positive feedback on our manuscript.

**Major Issues**

None

**Minor Issues**

1. Fig. 1E shows a schematic of a patients infected with S. typhimurium. This creates an impression that the experiment was performed in humans, please drop it.

We thank the reviewer for pointing this ambiguity. We corrected the figure to avoid confusion.

2. Fig. S1 shows a schematic depicting the experimental approach, please note this in the text.

We replaced “(Fig. S1A)” by “(experimental approach is depicted on Fig. S1A)”. We did the same for Fig. S1B and S1C.

3. Fig. 2 shows the level of nitrate, please explain how this represents RNS. Nitrate is virtually absent in Nos2- cells, but the difference in wild type cells +/- INF-g used throughout this work is modest, please explain.

To clarify the link between nitrite quantification and RNS production by macrophages, we added: “Quantification of nitric oxide production by macrophages was achieved by quantifying its stable byproduct nitrite in the infection medium.” in the figure legend of Fig. S2.

In our experimental setup, prestimulation with IFN-γ significantly increased the production of macrophage-derived RNS by 50% (from 30 uM to 45 uM, Fig. S1A). It is noteworthy that by essence in our assays macrophages are already stimulated by exposure to *Salmonella* (and the accompanying LPS). Stimulating macrophages with IFN-γ *in addition to the LPS stimulus*, results in this 50% increase. We have recently shown that this increase is sufficient to significantly impact the number of bacteria displaying intoxication of their TCA cycle (1).

4. The authors note that the number of persisters correlates tightly with the level of RNS, but such a correlation is not documented experimentally. One can make a qualitative assumption that RNS correlates with persisters, but “tightly” implies a quantitative measurement. Either provide the measurement or rephrase.

We corrected it in the manuscript and removed “tightly” to avoid any confusion.

5. Fig. 3D, please add a notation to the y axis to specify what is being measured; currently, it states “fluorescence”, not clear of what.

We replaced “Fluorescence intensity” by “Fluorescence intensity of Hoechst 33342”. For consistency, we also replaced y axis on Fig. 3F.

**Reviewer #2**

Host-derived reactive oxygen and nitrogen species (RNS) help control intracellular bacterial pathogens but can also antagonize antibiotic efficacy through collapsing bacterial TCA cycle and promoting persister cell formation. Enhanced tolerance to multiple different antibiotics under stressful conditions such as the intracellular niche has been demonstrated across a range of bacterial pathogens, including Salmonella. In this study, Ronneau and colleagues observed that intracellular Salmonella was more susceptible to ciprofloxacin than cefotaxime, and its tolerance to ciprofloxacin was not responsive to the RNS level. The authors hypothesize that host-derived RNS facilitates ciprofloxacin killing of intracellular Salmonella by suppressing bacterial respiration and the AcrAB–TolC efflux pump, causing the accumulation of ciprofloxacin in the bacteria. Mutants with defective efflux pump (ΔacrB, ΔtolC) and TCA cycle (ΔsucB) rendered reduced survival rates of intracellular bacteria after ciprofloxacin treatment. Extracellular bacteria exposed to exogenous RNS exhibited reduced efflux rate as reflected by the higher Hoechst fluorescent signal. Lower Hoechst efflux was also observed in the ΔacrB and ΔsucB mutants.

**Major Issues**

1.Active efflux pumps have been shown to reduce ciprofloxacin killing in planktonic Salmonella culture. The authors nicely demonstrated that the AcrAB–TolC efflux pump also contributed to bacterial survival under ciprofloxacin inside macrophages. However, with the existing data, it may be difficult to reach the conclusion that host-derived RNS was causing the inactivation of bacterial efflux pump and the enhanced ciprofloxacin killing in macrophages. If the host-derived RNS was the major factor, a higher survival rate should be observed in Nos2 knock-out macrophages compared to the wild-type macrophages, while no difference in the survival rates among Nos2 knock-out and wild-type macrophages with and without IFN-gamma was detected, suggesting that the better killing with ciprofloxacin was RNS-independent. Although the authors demonstrated that exogenous RNS could interfere with the efflux in extracellular bacteria, host-derived RNS may not be the reason for the better efficacy of ciprofloxacin.

As accurately pointed out by the reviewer, the absence of differential killing in the absence (*nos2-/-*) and presence of high levels of host RNS (IFN-γ-stimulated macrophages) indicates that RNS **do not** impact the overall efficacy of ciprofloxacin itself. We fully agree with the reviewer on this aspect and we do not intend to make such claim in our work.

In our previous studies, we showed that β-lactams only efficiently kill growing bacteria during infection (2–4). In contrast, ciprofloxacin is active against some, though not all, non-growing bacteria (2,5). Our current study sheds light on why not all non-growing bacteria surviving β-lactam treatment survive ciprofloxacin inside host cells. According to our findings, non-growing bacteria intoxicated by host RNS are unable to maintain an active efflux machinery. Consequently, they only survive cefotaxime but not ciprofloxacin. In contrast, persisters that are RNS-independent (observed in *nos2-/-* macrophages), can still survive ciprofloxacin treatment as they maintain an active efflux machinery (WT strain shown on Fig. 3F). Since RNS-dependent persisters are sensitive to ciprofloxacin, the levels of persisters surviving ciprofloxacin are comparable in the absence (*nos2-/-*) or presence of high RNS levels (IFN-γ-stimulated macrophages). This model is described more in details on Fig. 4B and we have added to the text to clarify it notably lines 298-303.

2.The differential susceptibility of intracellular Salmonella to ciprofloxacin and cefotaxime could be resulted from their ability to penetrate mammalian cell membranes and accumulate in macrophages. The authors may want to address if the intracellular bacteria were exposed to a sufficient and comparable amount of the two antibiotics in the intracellular niche.

The reviewer is correct; the concentration of antibiotics is influenced by their ability to enter mammalian cells. For instance, we observed an increase in the minimum inhibitory concentration (MIC) for ciprofloxacin when working within macrophages (See below; MIC around 0.1 ug/ml; the red star represents our working concentration) compared to *in vitro* conditions (0.02 ug/ml). Similarly, we have previously shown that although the MIC for cefotaxime is higher for bacteria inside macrophages, cefotaxime retains a strong bactericidal activity in our experimental conditions (2–4,6). For both antibiotics, we opted for a concentration significantly higher than the MIC (between 50-100x) that resulted in the killing of at least 99% of the population of intracellular *Salmonella* after 24 hours. Notably, surviving cells recovered after antibiotic treatment are exclusively non-growing bacteria; indicating *complete clearance of* growing bacteria (2–4,6). Furthermore, we noted similar killing effects for cefotaxime and ciprofloxacin in nos2-/- macrophages, supporting that bacteria were exposed to a sufficient and comparable amount of both antibiotics in our experimental setup (Fig. 1C).

**Minimal Inhibitory Concentration (MIC) and dose-dependent killing of ciprofloxacin in bone-marrow macrophages.** 24 h ciprofloxacin survival of WT *Salmonella* in WT Mφ normalized to values after 30 min internalization. Error bars depict means and standard deviation (SD); n = 3. The red star represents the working concentration used in the manuscript.

3.It may not be surprising that ΔacrB and ΔsucB mutants extracted from Nos2 knock-out (low RNS) macrophages had suppressed Hoechst efflux as shown in Figure 3F. However, to probe if RNS really impacts the efflux in the intracellular bacteria, the comparison between bacteria extracted from Nos2 knock-out (low RNS) and stimulated (high RNS) macrophages would be needed. The authors could also use efflux pump inhibitors to probe the efflux activity in RNS-high and RNS-low macrophages.

We thank the reviewer for their comment and agree this experiment would improve our work. To that effect, we performed efflux assays in bacteria released from Nos2-/- and IFN-γ-stimulated WT macrophages. Presence of RNS limited the efflux activity of cefotaxime persisters extracted from IFN-γ-stimulated WT macrophages (new Fig. S3B, lines 227-229), which supports our model further.

**Minor Issues**

1.In Figure 1E, data of Nos2 knock-out macrophages infected with the clinical isolates after ciprofloxacin treatment should be included for comparison.

We thank the reviewer for their comment. We added the requested data in Fig. 1E to allow the comparison.

2. The inconsistent intracellular survival rates of the acrB mutant under ciprofloxacin in Figure 2C and Figure 3G were noticed.

While we observed a difference in killing between panel 2C and 3G for the *acrAB* mutant, it is noteworthy that the strain presented in Fig. 3G contained the empty pCA24N vector. For reasons not fully understood at this point, the presence of the empty vector potentiate ciprofloxacin activity in the *acrAB* mutant.

**Reviewer #3**

The manuscript by Ronneau S. et al. explores the mechanism underlying a recent observation, that reactive nitrogen species (RNS) sensitizes Salmonella to fluoroquinolones. They show RNS targeting of the TCA cycle results in reduction of proton-motive force, preventing ciprofloxacin efflux from the bacterial cell. While ciprofloxacin accumulation was not measured directly, the authors utilized multiple approaches to detect efflux activity and correlate the reduction in efflux activity with ciprofloxacin sensitivity. They also utilized a complemented strain to show altered sensitivity is due to efflux. They incorporated clinical isolates into experiments as well, to show the relevance of these pathways for clinical strains.
The findings here provide an important advance in our understanding of antibiotic tolerance, and combating antibiotic tolerance cells, and the authors systemically explore several different aspects of their model through each Figure. The experiments are well-designed, and strongly support the authors conclusions. I have one suggestion for an additional experiment below with the acrAB complemented strain that I think could strengthen their model further, however the authors have shown the more critical complementation of the survival defect with this strain. This manuscript is extremely well-written, and I only have minor comments below:

We thank the reviewer for their very positive feedback on our manuscript.

**Major Issues**

Since the authors have generated a complemented strain (∆acrB + pacrAB, in Fig. 3G), it would also be important to show this strain no longer has increased accumulation of H33342. It was great to see the authors utilize this strain to complement the survival phenotype, but I think also showing the impact on H33342 accumulation should be included.

As suggested by the reviewer, we conducted an efflux assay with the complemented strain (∆acrB + pacrAB). We observed that the presence of pacrAB restored efflux in the *acrB* mutant and have added this to new Fig. S3A (line 210).

**Minor Issues**

• Fig. 2E: The GFP intensity quantification is described as ‘translational activity’, but really would be a combination of transcription, translation, fluorescent protein folding, and fluorophore maturation. I think a term like ‘Induction capacity’ or ‘Induction ability’ might more accurately reflect GFP intensity here.

The reviewer is correct; translation is not the sole parameter influencing this assay. We have replaced "Translational activity" with the broader term "Activity” throughout.

• Fig. 4: The model described here was cited briefly once in the text, and could have been discussed in more detail. Some discussion is already present, and filling in additional ‘Fig. 4’ references in the text would reinforce this by drawing the reader to the Figure. The authors should also consider adding a couple of additional sentences to reinforce some of the ideas in Fig. 4B, particularly the heterogeneity of efflux expression that is depicted here.

We thank the reviewer for their comment. We have now cited our model in lines 277, 300, and 303. Additionally, we have seized the opportunity to strengthen the discussion on the heterogeneity in efflux activity, as suggested by the reviewer (lines 298-303).

• Lines 219-220, Fig. 3: The concentrations of H33342 listed in the Results text (0, 0.2, 1, 2) don’t match the concentrations shown in Fig. 3F (0, 0.2, 0.5, 1). H33342 is written as ‘H33345’ in Fig. 3D and 3F.

We thank the reviewer for spotting this oversight. This has been corrected.

• Minor: line 262, missing ‘damage’ after DNA?

In a previous study (3), we demonstrated that antibiotic persisters accumulate DNA during infection. This finding helps to explain why drugs targeting DNA replication, such as ciprofloxacin, can cause damage to these non-growing cells.

• Minor: typo line 173, ‘onlybe’

Thanks.

**REFERENCES**

1.	Ronneau S, Michaux C, Helaine S. Decline in nitrosative stress drives antibiotic persister regrowth during infection. Cell Host Microbe. 2023;31(6):993-1006.e6.

2.	Helaine S, Cheverton AM, Watson KG, Faure LM, Matthews SA, Holden DW. Internalization of Salmonella by Macrophages Induces Formation of Nonreplicating Persisters. Science 343, 204–208.

3.	Hill PWS, Moldoveanu AL, Sargen M, Ronneau S, Glegola-Madejska I, Beetham C, et al. The vulnerable versatility of Salmonella antibiotic persisters during infection. Cell Host Microbe. 2021;29(12):1757-1773.e10.

4.	Michaux C, Ronneau S, Giorgio RT, Helaine S. Antibiotic tolerance and persistence have distinct fitness trade-offs. PLoS Pathog. 2022;18(11):e1010963.

5.	Keren I, Kaldalu N, Spoering A, Wang Y, Lewis K. Persister cells and tolerance to antimicrobials. FEMS Microbiol Lett. 2004;230(1):13–8.

6.	Stapels DAC, Hill PWS, Westermann AJ, Fisher RA, Thurston TL, Saliba AE, et al. Salmonella persisters undermine host immune defenses during antibiotic treatment. Science 362, 1156–1160.
